# Supplementary figures and images for: Catching the Big Fish in Big Data: A Meta-Analysis of Zebrafish Kidney scRNA-Seq Datasets Highlights Conserved Molecular Profiles of Macrophages and Neutrophils in Vertebrates
Source: Biology (Basel). 2024 Sep 27;13(10):773. doi: 10.3390/biology13100773 (PMC11505477; doi:10.3390/biology13100773)

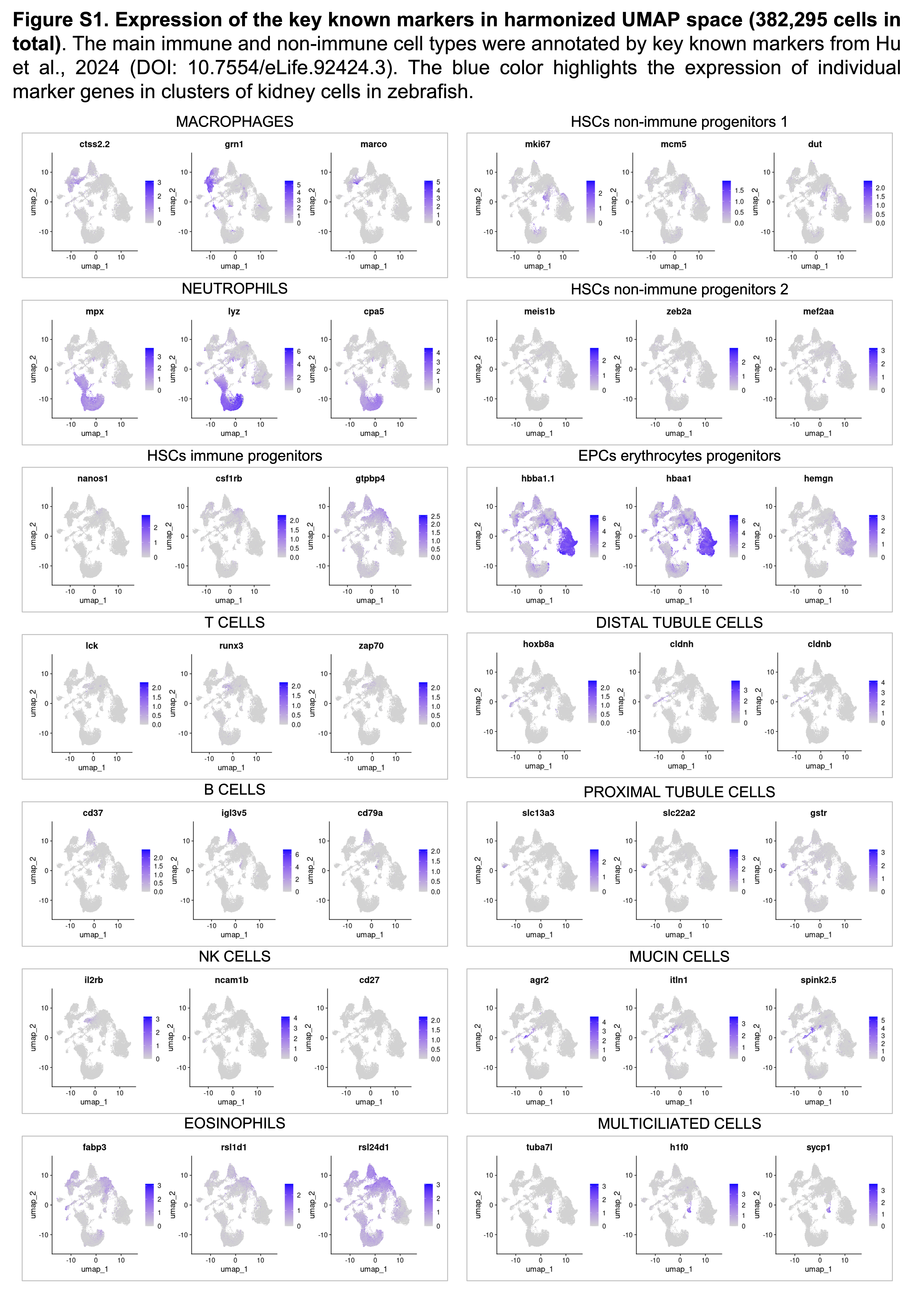

Supplement: Supplementary file 1 [file biology-13-00773-s001.zip › Zebrafish_kidney_MDPI_Supplementary_Files_review2/S1._Initital_analysis.Markers_of_all_cell_types.png]

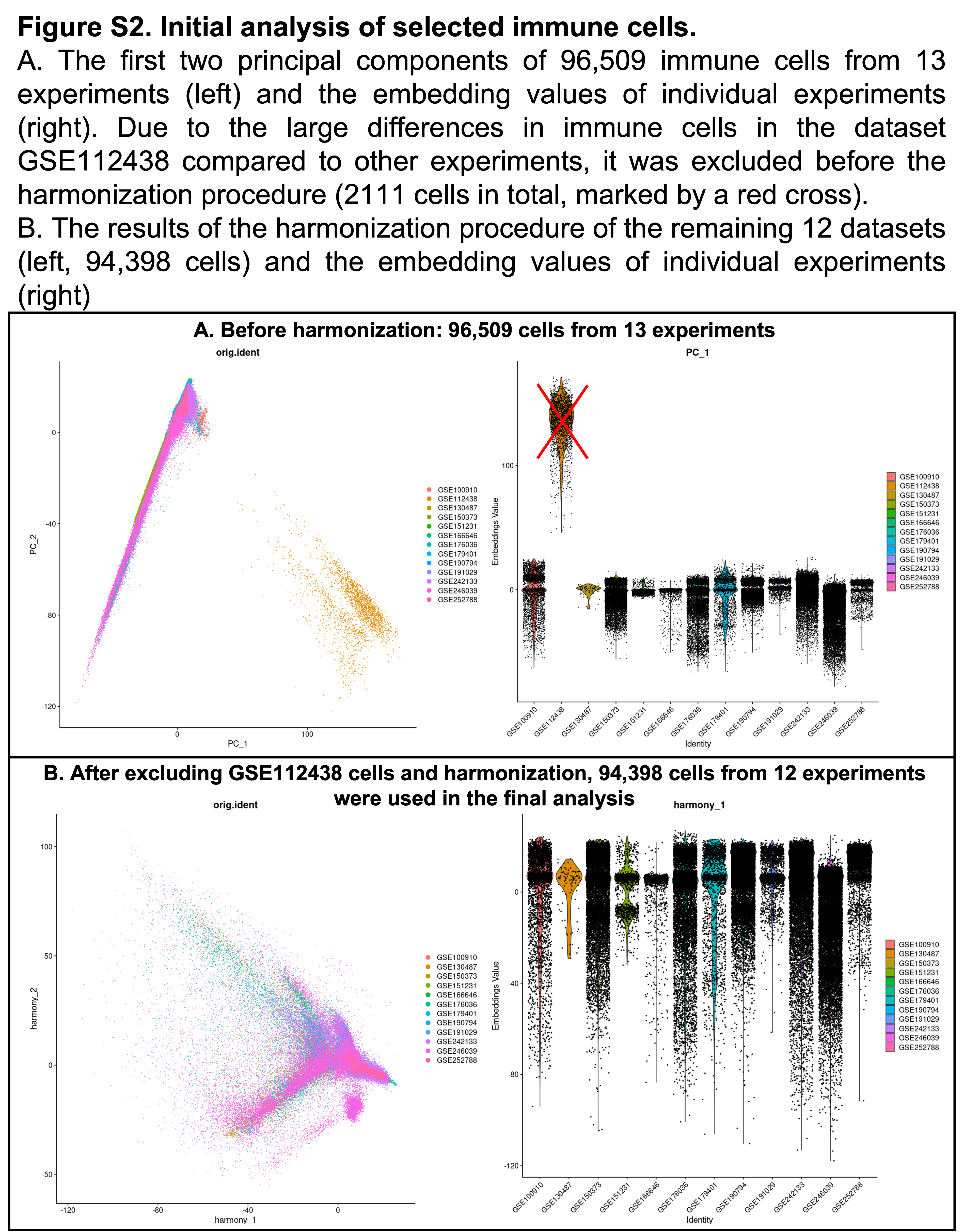

Supplement: Supplementary file 1 [file biology-13-00773-s001.zip › Zebrafish_kidney_MDPI_Supplementary_Files_review2/S2._Selected_immune_cells.png]

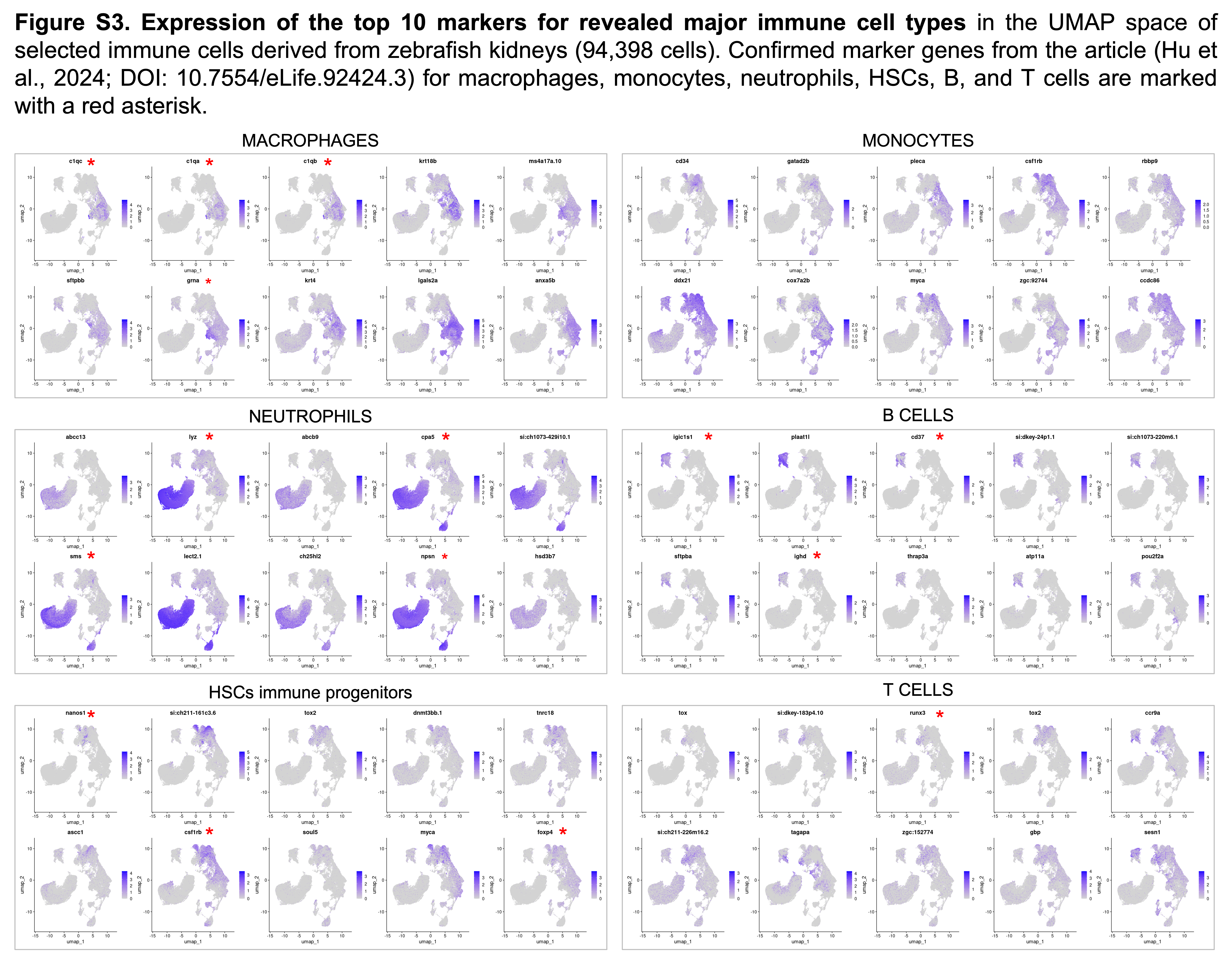

Supplement: Supplementary file 1 [file biology-13-00773-s001.zip › Zebrafish_kidney_MDPI_Supplementary_Files_review2/S3._Top_markers_of_immune_cell_types.png]
